# The Mechanism of CD8^+^ T Cells for Reducing Myofibroblasts Accumulation during Renal Fibrosis

**DOI:** 10.3390/biom11070990

**Published:** 2021-07-05

**Authors:** Min Gao, Jing Wang, Jianghua Zang, Yina An, Yanjun Dong

**Affiliations:** College of Veterinary Medicine, China Agricultural University, Beijing 100193, China; s20193050710@cau.edu.cn (M.G.); wangjing98@cau.edu.cn (J.W.); jianghuaz@cau.edu.cn (J.Z.); b20203050369@cau.edu.cn (Y.A.)

**Keywords:** fibroblast, myofibroblast, embryonic origin, cell diversity, development, extracellular matrix homeostasis, inflammation, renal fibrosis

## Abstract

Renal fibrosis is a hallmark of chronic kidney disease (CKD) and a common manifestation of end-stage renal disease that is associated with multiple types of renal insults and functional loss of the kidney. Unresolved renal inflammation triggers fibrotic processes by promoting the activation and expansion of extracellular matrix-producing fibroblasts and myofibroblasts. Growing evidence now indicates that diverse T cells and macrophage subpopulations play central roles in the inflammatory microenvironment and fibrotic process. The present review aims to elucidate the role of CD8^+^ T cells in renal fibrosis, and identify its possible mechanisms in the inflammatory microenvironment.

## 1. Introduction

Composed of interstitial fibrosis and glomerulosclerosis, kidney fibrosis is frequently observed in the final stage of chronic kidney disease (CKD) and is considered a common parameter of different types of CKD. The principal pathologic characteristics and central events of renal fibrosis are renal interstitial fibroblast activation and myofibroblast expansion in obstructive kidney disease and the anomalous and excessive deposition of the extracellular matrix, pathologies that give rise to the destruction of normal renal tubules and interstitial structures. Various stimuli and injuries (including: Ang II, high levels of glucose, hypoxia, ischemia, endo or exogenous nephrotoxins and immune molecules) can induce tissue cellular damage and the expression of relevant molecular products [1], which are considered to be crucial triggers for inflammation after acute kidney injury (AKI) [2]. Following injury, associated inflammatory cells are recruited to the injured site by the concentration gradients of chemotactic factors [3], including neutrophils, lymphocytes, monocytes/macrophages, dendritic cells, and mast cells. In the process, the recruitment of immune cells is favored by the upregulation of adhesion molecules secreted by diverse types of cells within the injured kidney [4]. This series of events produces a high concentration of local cytokines and build up a sustained inflammatory microenvironment, and then primes fibroblasts and myofibroblasts to undergo activation and expansion, eventually leading to renal fibrogenesis and extracellular excessive matrix (ECM) accumulation and deposition. Myofibroblasts and fibroblasts are the principal effector cells for ECM production. The ECM is a highly dynamic structure that acts as a support scaffold for kidney parenchymal cells. The balance between deposition and degradation of ECM is necessary to maintain tissue homeostasis, whereas break of this balance causes renal fibrosis.

Inflammation normally serves as a protective process that it eliminates damage and promotes kidney repair. However, unresolved inflammation induces and initiates renal fibrosis. In response to chemokines released by injured resident renal cells, heterogeneous T cells are attracted to the injured kidney in a model of renal fibrosis [5,6,7,8,9]. To date, the roles of T cells have been studied exclusively by using various depletion techniques [10,11,12,13]. Increasing studies showed that γδ T cells, Th17 cells and CD4^+^ T cells exert a profibrotic effect on injured kidney [11,14], whereas Tregs protect the kidney against injury and fibrosis [15]. Of note, Tregs can kill the activated immune cells through granzyme B or Fas-FasL [16,17,18], and control phenotype transition macrophages to prevent inflammation and promote tissue repair [19,20,21]. The role of CD8^+^ T cells in renal inflammation and fibrosis is less defined. Here we show that infiltration of CD8^+^ T cells exists throughout the entire process of renal inflammation and fibrosis and plays a pivotal role in regulating the accumulation of myofibroblasts in injured kidney.

## 2. Myofibroblast Accumulation in Renal Fibrosis

Myofibroblasts are involved in numerous fibrotic and scarring diseases following injury. Utilizing a transgenic reporter mouse expressing enhanced green fluorescent protein (GFP) that is regulated by the collagen type I alpha 1 (coll1a1) promoter, Lin et al. identified the origins of coll1a1-producing cells in the kidney. The results showed that cells expressing α-SMA were approximately 75% of GFP positive cells [22]. Myofibroblasts are thus shown to be a principal source of ECM production in these lesions, while fibroblasts are also the major contributor of ECM of connective tissue. Another important function of myofibroblasts and fibroblasts is the homeostatic maintenance of the ECM of the kidney where they reside. Fibroblasts produce MMPs and TIMPs, which regulate the degradation and deposition of ECM. During renal remodeling and inflammation resolution, the expression of MMPs and TIMPs changes the equilibrium to favor ECM deposition over a matrix-degrading environment, and subsequently myofibroblasts are removed by apoptosis [23]. Remaining fibroblasts exhibiting a quiescent state may come from reverted myofibroblasts and peripheral blood fibrocytes [24].

**Figure 1:**
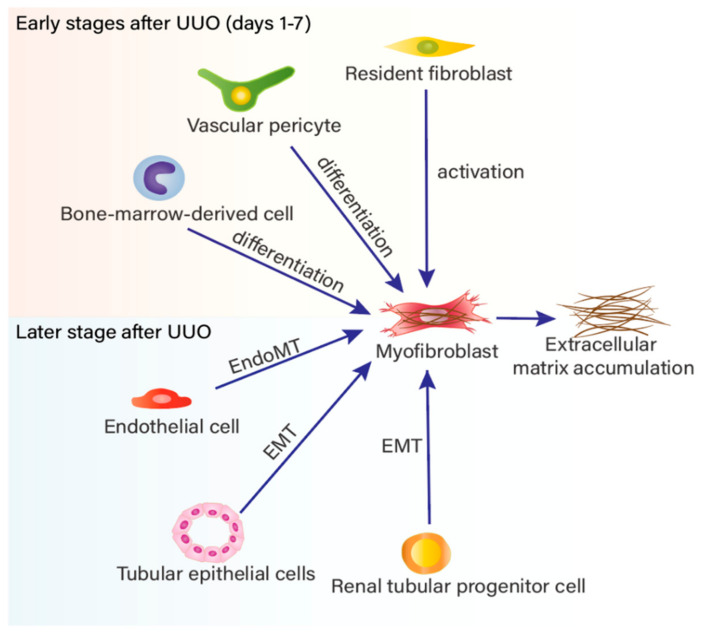
The cellular origins of myofibroblasts in renal fibrosis.

The exact origin of myofibroblasts during renal fibrosis is highly controversial. Considerable research on the origin of myofibroblasts during renal fibrosis has utilized lineage tracing and marker location technologies and finally suggested that myofibroblasts may derive from diverse progenitor cells (Figure 1). Currently, multiple identified origins include the activation of resident fibroblasts [25,26], proliferation or/and differentiation of pericytes [22,27,28], epithelial-mesenchymal transition (EMT) [29,30,31], endothelial-mesenchymal transition (Endo-MT) [32,33], and bone marrow-derived cells [10,34,35,36,37,38,39,40], of which resident fibroblasts activation is the predominant contributor [26]. A further and classical study showed that 35% of myofibroblasts in a unilateral ureter obstruction (UUO) model of renal fibrosis were derived from the bone marrow [41]. It has been recently demonstrated that the proportion consists of the differentiation of mesenchymal stem cells (MSCs) [42], fibrocytes-to-fibroblast transition [36,37] and macrophage-to-myofibroblast transition (MMT) [39,43]. In addition, there is a contribution of EMT (5%), Endo-MT (10%), and interstitial fibroblast (50%). Common markers of pericytes, such as PDGFR-β and NG2, are not entirely specific to pericytes, so whether the pericytes identified and interstitial fibroblasts are the same population remains to be elucidated [44]. In addition, resident fibroblasts, bone marrow-derived cells and perivascular pericytes are likely to be the major sources of activated fibroblasts and myofibroblasts during the first hours after UUO [22], whereas EMT-derived and Endo-MT-derived myofibroblasts are not thought to appear until seven days after UUO. It is important to note that the collagen matrix is susceptible to proteolysis in the early stage of renal fibrosis. Therefore, in the early stage, fibrosis is reversible and prevention of fibrosis could be an effective part of treatment. Another important issue is that MSC-based cyto-therapy is attracting increasing attention due to the capacity of MSCs to serve to protect against fibrosis in renal disease [45].

Notably, MMT and fibrocyte-to-fibroblast transition evidence that myofibroblasts transition from bone marrow-derived cells. MMT could be a pivotal checkpoint for the progression of unresolved inflammation into pathogenic kidney fibrosis. Another important issue is fibrocyte-to-fibroblast transition in renal fibrosis pathogenesis. A longstanding question in this field is the relationship between bone marrow-derived fibrocytes and collagen-producing macrophages. Due to the lack of exclusive markers for these cells, identifying fibrocytes from monocytes, macrophages, fibroblasts and myofibroblasts is especially problematic. Recent observations provide a further impetus for elucidating how fibrocytes could be distinguished by Gr1^+^CD115^−^Collagen I^+^ phenotype from the other cells, and are thought to develop in the bone marrow from myeloid precursor cells and migrate to injury sites where they differentiate into fibroblasts and promote renal fibrosis [35,37,46].

The process of renal fibrosis is mainly regulated at the level of gene transcription in response to multiple extracellular fibrotic signals. Vital growth factors include transforming growth factor β (TGF-β), platelet-derived growth factor (PDGF), basic fibroblast growth factor (FGF2), connective tissue growth factor (CTGF), epidermal growth factor (EGF) and angiotensin II. TGF-β1 has been deemed to serve as the predominant mediator of the pathology of renal fibrosis, whereas interferon-γ (IFN-γ), hepatocyte growth factor (HGF) and bone morphogenetic protein 7 (BMP-7) inhibit the production of matrix components primarily by counteracting TGF-β1 action [47,48,49,50,51,52,53]. In addition, myofibroblasts and fibroblasts may also secrete inflammatory factors that contribute to development of an inflammatory microenvironment [54,55], such as TGF-β1, IL-1β, interleukin-6 (IL-6), IL-13, IL-33 and chemokines [56,57]. Thus, the inflammatory microenvironment plays a crucial role in inducing myofibroblast accumulation in renal fibrosis.

## 3. Varied Phenotypes of CD8^+^ T Cells in Renal Inflammation and Fibrosis

In the inflammatory microenvironment, a variety of pro-inflammatory factors such as TNF-α, IFN-γ, IL-6, IL-1β, IL-23, IL-17, C3, C5a, C5b and anti-inflammatory mediators including IL-4, TGF-β, IL-10, heme oxygenase 1, resolvins, and protectin D1 are secreted by resident and recruited cell populations. The expression and effects of these inflammatory factors exhibit the characteristics of time and concentration dependence, which are pivotal determinants of the injury and repair phases [58]. The equilibrium between pro- and anti-inflammatory mediators has a significant impact on the extent of tissue injury and repair, decides the ongoing direction between unresolved inflammation and the resolution phase, and eventually leads to kidney fibrosis or rehabilitation. From a clinical point of view, it implies a better understanding of controlling the balance in the injured kidney, which may give rise to more associated intervention approaches targeting fibrosis and scarring.

During inflammation in the early stage of renal fibrosis, CD8^+^ T cells play a pivotal role in the balance of the inflammatory microenvironment. In the response to acute and chronic kidney injury, the recruitment and activation of T lymphocytes typically precedes the influx of macrophages into the injured kidneys [12]. Meanwhile, the bone marrow increases the production and release of monocytes into the peripheral circulation [59]. CD8^+^ T cell accumulation increases from day 1 and reaches a peak at day 5 after UUO injury [60]. It is worth noting that the absence of CD8^+^ T cells did not influence the expression of MCP-1, MIP-1α, MIP-1β and RANTES in UUO-treated mice [13], because other T cells subpopulations could compensate for the absence. CD4^+^ T cells and CD8^+^ T cells produce multiple chemokines to induce monocyte recruitment, such as KC, MCP-1, MIP-1α, MIP-1β and RANTES [12,54]. In response to colony-stimulating factor 1 (CSF1), recruited monocytes can proliferate in the kidney, which amplifies the inflammatory response [61,62,63]. IFN-γ and TNF-α secreted by CD8^+^ T cells initiate relevant signaling pathways to induce macrophage polarization towards the M1 phenotype which can produce a higher level of proinflammatory cytokines, such as TNF-α and IL-1β, IL-6 and chemokines like MCP-1 [64]. CD8^+^ T cells and Th2 cells secrete IL-4 and IL-13, subsequently inducing the infiltrated M1 phenotype to change into the profibrotic M2 macrophages involved in kidney repair and regeneration, which are a major source of TGF-β and anti-inflammatory cytokines, such as IL-10, IL-1 [65]. The ongoing activation of TGF-β triggers macrophage transition from the M2a phenotype to myofibroblasts within the injured kidney (MMT) [38,39,66,67,68,69]. It is of note that M2 macrophages are the principal effector cells for TGF-β1 production which play a crucial role in the activation of intrinsic fibroblasts and accumulation of myofibroblasts through driving EMT [53,70,71,72,73], Endo-MT [74,75,76], fibrocyte-to-fibroblast transition [10,35,36,37], macrophage-to-myofibroblast transition (MMT) [39,43,66,77] and pericyte proliferation [22,27]. Additionally, M2 macrophages secrete IGF-1 and PDGF to maintain the survival of myofibroblasts and facilitate the proliferation and activation of resident fibroblasts [78,79]. The exhibition of varied phenotypes of CD8^+^ cells in inflammation and fibrosis leads researchers to identify different subsets of CD8^+^ cells in renal fibrosis and uncover their function.

## 4. The Role of CD8^+^ T Cells in Renal Fibrosis

A 2010 study found no significant effect for CD8^+^ T cells in renal fibrosis [9]. Tapmeier et al. have shown that CD4^+^ T cells but not CD8^+^ T cells restore the severity of UUO-induced fibrosis. After reconstitution of RAG^−/−^ mice with CD4^+^ T cells or CD8^+^ T cells in an obstructed kidney model, the reconstitution with CD4^+^ T cells restores fibrosis, but reconstitution of RAG^−/−^ mice with CD8^+^ T cells does not significantly influence renal fibrosis. Considering reconstituted CD8^+^ T cells may lack interaction with CD4^+^ T cells to elicit their effect in vivo, Tapmeier et al. hypothesized that CD8^+^ T cells play a potential antifibrotic role in UUO model.

Recent evidence from our group and others supports the idea that increased infiltration of CD8^+^ T cells attenuates renal fibrosis in mice. CD8 deficiency exacerbates renal fibrosis, whereas adoptive transfer of CD8^+^ T cells into CD8 KO mice decreases renal fibrosis in UUO-treated mice [13,60]. Zhao et al. suggest that IFN-γ-producing CD8^+^ T cells accumulate in the obstructive kidney to protect against UUO-induced renal fibrosis [80]. Astaxanthin induces the recruitment and infiltration of CD8^+^ T cells by upregulating CCL5. Data from Prediman et al. reveals that depletion of CD8^+^ T cells increases renal fibrotic gene expression in angiotensin II (AngII)-infused apoE^−/−^ mice. CD8^+^ T cells modulated by p210 vaccine (apoB-100 related peptide) could ameliorate AngII-induced hypertension and renal fibrosis [81]. These findings point to a potential anti-fibrotic and renal-protective role of CD8^+^ T cells and underpin the need to define the role and understand the mechanism of CD8^+^ T cells in renal fibrosis. Data from Liu et al. suggests that CD4^+^ T lymphocytes, especially Th2 cells, contribute to the progress of renal fibrosis [11]. Several previous studies suggested that IL-2, TNF-a, IFN-γ and IL-12 could inhibit the development of human and murine fibrocytes, whereas IL-4, IL-13 promoted the differentiation of fibrocytes from their progenitors [10,82,83]. CD4^+^ T cells isolated from obstructed kidney of CD8 KO mice promote more monocyte-to-fibroblast transition in vitro than CD4^+^ T cells isolated from obstructed kidneys of WT mice. To determine whether CD8^+^ T cells interact with CD4^+^ T cells to exert their effect during fibrosis, CD4^+^ T cells were depleted in CD8 KO mice or CD8^+^ T cell–reconstituted CD8 KO mice by using a monoclonal CD4 Ab to examine fibrotic areas and fibrosis-related protein levels in UUO kidneys [13]. Consistent with Tapmeier’s results, we found that CD8^+^ T cells do not significantly affect fibrosis in the absence of CD4^+^ T cells. Depletion of CD4^+^ T cells reduced fibrosis in the obstructed kidney, regardless of the presence or absence of CD8^+^ T cells.

## 5. IFN-γ-Producing CD8^+^ T Cells in Renal Fibrosis

Several previous studies have also suggested that CD8^+^ T lymphocytes could play a role in the generation of Th1 immunity and in the inhibition of Th2 response [84,85,86]. Our previous report suggested that CD8 deficiency increases fibrosis by promoting CD4^+^ T cell differentiation to Th2 [13]. We isolated CD4^+^ T cells from the obstructed kidneys of WT and CD8 KO mice and examined mRNA expression of Th1 markers IFN-γ and T-bet and of Th2 markers IL-4 and GATA3. IFN-γ and T-bet mRNA expression decreased, whereas IL-4 and GATA3 expression increased in CD4^+^ T cells isolated from CD8 KO UUO kidneys compared with WT CD4^+^ T cells. These data indicate that depletion of CD8^+^ T cells promotes differentiation of CD4^+^ T cells to a Th2 phenotype.

**Figure 2:**
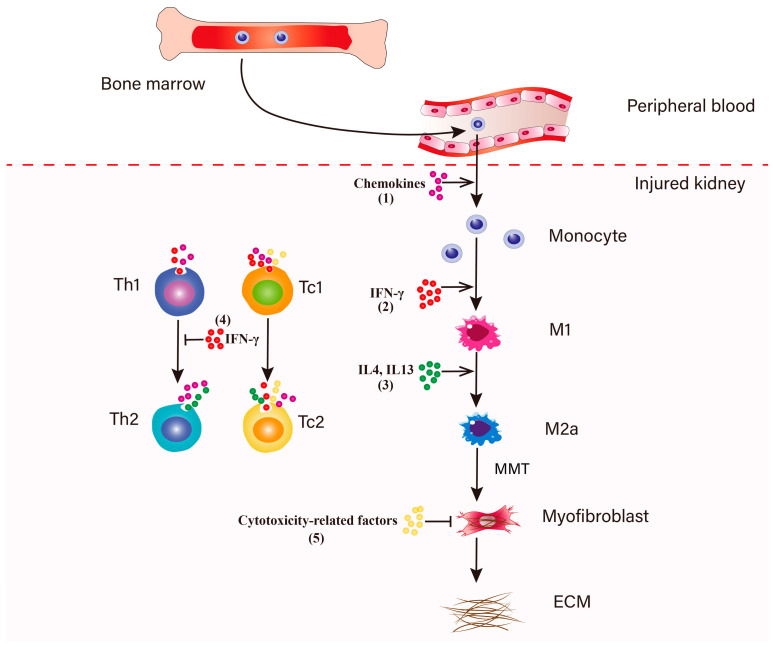
Regulation of macrophage-to-myofibroblast transition. (1) In response to kidney injury, the bone marrow increases the production and release of monocytes into the peripheral blood. Circulating monocytes are recruited to the injured kidney, a process that is dependent on the chemokines partly secreted by resident T lymphocytes and macrocytes [118,119]. Recruited monocytes can proliferate in the kidney to amplify the inflammatory response. (2) IFN-γ produced by Th1 and CD8^+^ T lymphocytes promote macrophages polarization towards a pro-inflammatory M1 phenotype [85,90]. (3) The infiltrated macrophages adopt an anti-inflammatory M2 phenotype in response to IL4 and IL13 which are expressed by Th2 and Tc2 cells [82]. The ongoing activation of TGF-β triggers macrophage transition from a M2a phenotype to myofibroblasts within the injured kidney (MMT) [38,39,66,67,68,69]. These MMT-derived myofibroblasts induce the accumulation and deposition of ECM and subsequently promote the development of renal fibrosis [38,39,43]. (4) During this process, two subsets of CD8^+^ T cells, Tc1 and Tc2 cells secrete IFN-γ to inhibit the differentiation of Th2 cells, which can prevent the Th2 cells-induced excessive polarization of M2a cells [34]. (5) Tc1 and Tc2 cells could secrete cytotoxicity-related factors to induce fibroblasts apoptosis, subsequently suppressing the expansion of myofibroblasts.

An early study demonstrated that IFN-γ secreted by natural killer cells (NK cells) inhibits the activation of fibroblasts to the myofibroblast phenotype and reduces renal fibrosis [87]. Consistently, Fariba et al. suggested the IFN-γ-peptidomimetic fibroferon targeted to PDGFβR-overexpressing myofibroblasts attenuates renal fibrosis [88]. Ryo et al. showed that interferon-γ enhances the anti-fibrotic effect of mesenchymal stem cells (MSCs) on experimental renal fibrosis, and it is through the secretion of prostaglandin E2 that MSCs can exert a protective effect on rats subjected to ischemia–reperfusion injury (IRI) and UUO [89]. In recent years, studies from Patrícia et al. suggest that, besides NK (through IFN-γ) and dendritic cells (through IL-12), CD8^+^ T lymphocytes, as another source of IFN-γ, likewise enhances CD4^+^ Th1 phenotype development [90]. IFN-γ favors Th1 cell differentiation of CD4^+^ T cells and inhibits Th2 cell differentiation [91,92]. Of note, CD8^+^ T cells produce a higher level of IFN-γ than CD4^+^ T cells after TCR activation [90]. Furthermore, IFN-γ exerts an anti-fibrotic effect on injured kidney via inhibiting fibroblast activation and proliferation and reducing collagen synthesis [93]. It may be important for improving the understanding of mechanisms and therapies of renal fibrosis to investigate the expressing characteristics of cytokines during the pathological process. Data from our previous study indicated the roles for CD8^+^ T cells in renal inflammation and fibrosis, where IFN-γ plays a key role in the CD8^+^ T cell regulatory mechanism in the pathological process [13]. We isolated CD8^+^ T cells from IFN-γ KO mice and injected them or WT CD8^+^ T cells into CD8 KO mice. The results showed that WT CD8^+^ T cells could reduce the differentiation of CD4^+^ T to Th2 cells and renal fibrosis, whereas reconstitution by IFN-γ KO CD8^+^ T cells did not impair fibrosis or transition of Th1 into Th2 phenotype. Consistently, some data by our group and others indicated that IFN-γ secreted by CD8^+^ T cells inhibits the transition of Th1 cell into Th2 cells, and thus decreased M2 macrophage polarization and macrophage-to-myofibroblast transition or monocyte-to-fibroblast transition, subsequently attenuating the extent of renal fibrosis (Figure 2) [11,13].

## 6. The Functions of CD8^+^ T Cell Subsets Which Identified by CD44, CD25 and CD62L on Myofibroblasts Accumulation in Renal Fibrosis

Currently, cytolytic CD8^+^ T effector cells are divided into two subpopulations according to cytokine secretion. Type 1 CD8^+^ T cells (Tc1) secrete IFN-γ, while type 2 CD8^+^ T cells (Tc2) mainly secrete IL-4 and IL-5 [94]. Although both effector cell subpopulations have been identified in human peripheral blood and in patients with various clinical disorders [95,96,97,98,99], their roles and effects on renal inflammation and fibrosis remain relatively undefined. We identified two CD8^+^ T cell subsets in a mouse model of UUO-induced renal fibrosis, Tc1 (CD44^+^CD25^−^CD62L^−^) and Tc2 (CD44^+^CD25^high^CD62L^low^), which exert diverse effects in building an inflammatory and profibrotic environment. During the early inflammation stage, Tc1 cells are the major population. Tc1 and Tc2 secrete chemokines that recruit macrophages. Additionally, Tc1 and Tc2 could increase the secretion of chemokines of infiltrated macrophages and fibroblasts. Following the development of fibrosis, CD8^+^ T cells transition into Tc2 phenotype, which secrete a higher level of IL-4 and IL-13 than Tc1 to induce the accumulation of M2 macrophages and myofibroblasts in the obstructed kidney (Figure 2). Our results showed that Tc1-treated macrophages activate fibroblasts to secrete inflammatory factors and produce excessive ECM. It is of note that Tc1 and Tc2 both secreted IFN-γ to inhibit the transition of CD4^+^ T cells to Th2 phenotype, and thus decreased the expression of IL-4 and IL-13 and the accumulation of M2a phenotypic macrophages and myofibroblasts [67,68,100]. Additionally, some studies demonstrated that Th1 cell subpopulations producing IFN-γ seemed to be related to the cytotoxic effect of CD8^+^ T cells [101].

## 7. Cytotoxic Effect of CD8^+^ T Cells on Fibroblasts

Cytotoxic T lymphocytes could kill cancer cells, infected cells and damaged cells. CD8^+^ T cells are cytotoxic in many fibrosis diseases, such as liver fibrosis [102], systemic sclerosis [103], lung fibrosis [104], wound healing [105] and tumor [106]. Some data show that T cells could induce fibroblast apoptosis [107,108,109,110]. However, whether their cytotoxic effect reduces renal fibrosis has not been well studied.

There are two direct manners for CTL-mediated cytotoxicity to target cells. Activated CD8^+^ T cells produce FAS ligand that interacts with FAS on the target cell, resulting in apoptosis of the target cells through the activation of caspase-8 and caspase-3. The other manner that CD8^+^ cytotoxic effector cells use is via the calcium-dependent release of specialized lytic granules including principally perforin and granzymes. Perforin polymerizes to form transmembrane pores in the target cell membrane, enabling the diffusion of granzymes into the cytosol, thus initiating apoptosis of the target cell through the activation of caspase-3 [111,112].

CD11c is a marker used to identify and characterize dendritic cell (DC) subsets [113]. In 1987, one study first reported that cytotoxic CD8^+^ T cells could express CD11c [114]. CD11c^+^CD8^+^ T cells exhibit potent cytotoxic effects in vitro and in vivo [115]. Chen et al. found that responding to bacterial infection, CD11c^+^CD8^+^ T cells constitute a heterogeneous population that can be further divided into CD11c^high^CD8^+^ T cells and CD11c^low^CD8^+^ T cells. The former could directly kill the activated CD4^+^ T cells both in vitro and in vivo, whereas the latter could secrete plentiful IFN-γ and induce the apoptosis of OVA-transfected EL4 (EG7) target cells through perforin [107]. Similarly, we separated CD11c^+^CD8^+^ T cells and CD11c^−^CD8^+^ T cells from the kidney in mice subjected to UUO, detected mRNA expression of cytotoxicity-related genes, such as perforin 1, granzyme A, granzyme B and FAS ligand in the two CD8^+^ T cells and identified which cell type had undergone apoptosis. The results indicated that CD8^+^ T cells were distributed around fibroblasts, and they are related to fibroblast apoptosis. CD11c^+^CD8^+^ T cells could express higher levels of the cytotoxicity-related genes than CD11c^−^CD8^+^ T cells. Of note, CD11c^+^CD8^+^ T cells play a major role in killing fibroblasts (Figure 2), although CD11c^−^CD8^+^ T cells could also induce fibroblast apoptosis. Unfortunately, we did not further identify how the two cells respectively exhibit cytotoxic effects on fibroblasts. More understanding of targeting the apoptosis of activated fibroblasts and myofibroblasts is required to develop therapeutic measures for extenuating renal fibrosis.

## 8. Some Limitations of Existing Research and Perspective

CD8^+^ T cells play a central role in the adaptive immune response. Activation of CD8^+^ T cells occurs through recognizing pathogen-derived peptide-major histocompatibility complex class I (pMHC-I) of antigen presenting cells (APCs) [116]. However, the mechanism of CD8^+^ T cell activation in renal fibrosis induced by sterile inflammation is not elucidated. The phenotype and function of CD8^+^ T cells changes with the inflammatory microenvironment. One limitation of current researches is that the balance of CD8^+^ T cells subsets in mice with renal fibrosis is unclear. How to ameliorate renal fibrosis by balancing CD8^+^ T cells subsets may be a question to consider. Data from Schumacher et al. demonstrate that a capacity to recognize autologous tumors is limited to approximately 10% of tumor-infiltrating CD8^+^ T cells [117]. Previous research validated that CD11c^+^CD8^+^ cells account for 58% of the CD8^+^ T cells population in obstructed kidney. The restricted characterization of both CD11c^+^CD8^+^ T cells and CD11c^−^CD8^+^ T cells populations by their basic phenotype is an additional limitation. Indeed, we do not confirm that these populations are all functional cytotoxic T cells, and fail to ascertain how many of these cells necessarily correlate with the number of cytotoxic T cells. CD11c^+^CD8^+^ T cells may express other markers and perform other functions. CD8^+^ T cells could kill tumor cells through the cytotoxic effect. We found that CD8^+^ T cells also exhibit cytotoxic effects on fibroblasts, but it is unknown whether the cytotoxic effect is specific. We hypothesized that TCR of cytotoxic T cells recognizes surrounding myofibroblasts which are in an over-activated and aging state. Single cell RNA-seq and mass spectrometry analysis may help to unmask these mysteries by comparing the genetic and functional heterogeneity of diverse CD8^+^ T cells subsets.

## 9. Conclusions

Our previous study revealed that CD8^+^ T cells participate in the entire renal inflammation and fibrosis process and reduce myofibroblasts accumulation during renal fibrosis in two ways: (1) IFN-γ-producing CD8^+^ T cells reduce the differentiation of CD4^+^ T cell into Th2 cells to control inflammation and fibrosis. (2) CD11c^+^CD8^+^ T cells in obstructed kidney could induce fibroblast apoptosis. It may be beneficial for the prevention and treatment of renal fibrosis to regulate the number and function of CD8^+^ T cells in different periods of fibrogenesis. More and further researches on the effect of CD8^+^ T cells for regulating renal inflammation and attenuating fibrosis will be necessary.
